# Designing and Managing Advanced, Intelligent and Ethical Health and Social Care Ecosystems

**DOI:** 10.3390/jpm13081209

**Published:** 2023-07-30

**Authors:** Bernd Blobel, Pekka Ruotsalainen, Mathias Brochhausen, Edson Prestes, Michael A. Houghtaling

**Affiliations:** 1 Medical Faculty, University of Regensburg, 93053 Regensburg, Germany; 2 eHealth Competence Center Bavaria, Deggendorf Institute of Technology, 94469 Deggendorf, Germany; 3 First Medical Faculty, Charles University of Prague, 11000 Staré Město, Czech Republic; 4 Faculty of Information Technology and Communication Sciences, Tampere University, 33100 Tampere, Finland; pekka.ruotsalainen@tuni.fi; 5 Department of Biomedical Informatics, University of Arkansas for Medical Sciences, Little Rock, AR 72205, USA; mbrochhausen@uams.edu; 6 Informatics Institute, Federal University of Rio Grande do Sul, Porto Alegre 90040-060, Brazil; prestes@inf.ufrgs.br; 7 IBM-Retired, Tucson, AZ 85750, USA; michael-h@acm.org

**Keywords:** health transformation, ecosystems, knowledge representation and management, architecture

## Abstract

The ongoing transformation of health systems around the world aims at personalized, preventive, predictive, participative precision medicine, supported by technology. It considers individual health status, conditions, and genetic and genomic dispositions in personal, social, occupational, environmental and behavioral contexts. In this way, it transforms health and social care from art to science by fully understanding the pathology of diseases and turning health and social care from reactive to proactive. The challenge is the understanding and the formal as well as consistent representation of the world of sciences and practices, i.e., of multidisciplinary and dynamic systems in variable context. This enables mapping between the different disciplines, methodologies, perspectives, intentions, languages, etc., as philosophy or cognitive sciences do. The approach requires the deployment of advanced technologies including autonomous systems and artificial intelligence. This poses important ethical and governance challenges. This paper describes the aforementioned transformation of health and social care ecosystems as well as the related challenges and solutions, resulting in a sophisticated, formal reference architecture. This reference architecture provides a system-theoretical, architecture-centric, ontology-based, policy-driven model and framework for designing and managing intelligent and ethical ecosystems in general and health ecosystems in particular.

## 1. Introduction

The paper at hand presents an extended version of the keynote provided to the pHealth 2021 conference [1], resulting in some inevitable similarities. This section introduces context, challenges and general solutions for transforming health and social care ecosystems.

For many years and everywhere around the globe, health and social care systems have been challenged by ongoing demographic changes towards aging, multi-diseased societies, the related development of human resources, health and social services consumerism, medical and biomedical progress, and exploding costs for health-related R&D as well as health services delivery. To overcome those problems, these systems must undergo transformations from traditional, hierarchical and regulated medicine towards an advanced health ecosystem for improving care quality and patient safety, but also efficiency and efficacy of care processes. This includes improved access to healthcare services for greater and autonomous active patient participation as well as improved decision making [2]. An ecosystem is the structural and functional unit of ecology where the living organisms interact with each other and the surrounding environment. It is the community of living organisms in conjunction with non-living components of their environment, interacting as a system [3]. The transformation towards personalized, preventive, predictive, participative precision medicine (5PM), supported by technology, is accompanied by important organizational and methodological paradigm changes, summarized in Table 1 and Table 2 as follows.

The described advancement of the care types from empirical to systems medicine finally results in transformed health ecosystems for ubiquitous personal health with the objectives being to provide individualized health services everywhere anytime, integrating all contributing actors and domains. This transformation must be supported by appropriate methodologies and technologies. The methodologies and technologies deployed to meet health transformation are shown in Table 3.

For designing, managing and implementing the described transformed health and social care ecosystems, new techniques and methods have to be deployed. Here, we have to mention mobile, bio-, nano- and molecular technologies, big data and analytics, virtual reality, learning algorithms as well as new computing technologies such as cloud, cognitive and edge computing. Furthermore, we also need appropriate policies and governance schemes to control the system’s behavior. An overview on the technologies and methodologies enabling 5PM ecosystems is presented in Table 4.

The intrinsic nature of technology enabling transformed health ecosystems requires a philosophical and especially ethical consideration of the approach [2] to be discussed in more detail in Section 2.3 and Section 3, respectively.

In the following section, we will introduce the underlying concepts of ecosystems and their management in more detail.

## 2. The 5PM Health Ecosystem

The aforementioned transformation of health systems aims at personalized, preventive, predictive, participative precision medicine (5PM), supported by technology. It considers individual health status, conditions, and genetic and genomic dispositions in personal social, occupational, environmental and behavioral contexts. In doing so, it transforms health and social care from art to science by fully understanding the pathology of diseases and turning health and social care from reactive to proactive.

The system represented by the subject of care and the processes analyzing and managing his/her health comprises all levels of granularity from elementary particles through atoms, molecules, cell components, cells, tissues, organs, bodies and communities, up to population. Regarding the functional or, in general, inter-relational aspects of that system, the relations comprise, e.g., quantum-mechanical effects in the nano-world, biochemical processes, interrelations based on classical physics and, finally, social interrelations in the macro-world.

**Figure 1:**
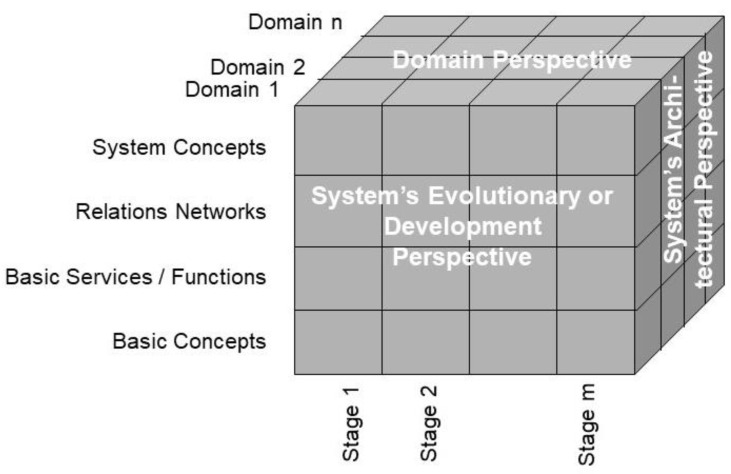
Generic model to represent ecosystems (after [11], modified).

For describing such an ecosystem, universal type theory and universal logics, formally represented using the Barendregt Cube [10], can be deployed. This approach can be advanced through system-theoretical and engineering principles by representing any ecosystem with its components, their functions and relations in the tree dimensions (Figure 1):

- The system’s architectural perspective, representing the system’s composition/decomposition or specialization/generalization;
- The system’s domain perspective, representing the involved domains and their actors;
- The system’s evolutionary or development perspective.

The described 5PM services require cooperation of many different and sovereign stakeholders from different policy domains in a multi-disciplinary approach including medicine, natural sciences, engineering, but also social, legal and political sciences and the entire systems sciences world (systems medicine, systems biology, systems pathology, etc.), performed through any type of principals (person, organization, device, application, component, object).

This requires the advancement of communication and cooperation among the business actors from different domains with their specific objectives and perspectives from data level (data sharing) to concept/knowledge level (knowledge sharing). Thereby, we have to recognize that they use different methodologies, terminologies/ontologies, education, skills and experiences.

The challenge is the understanding and the formal as well as consistent representation of the world of sciences and practices, i.e., of multidisciplinary and dynamic systems in variable context, for enabling mapping between the different disciplines, methodologies, perspectives, intentions, languages, etc., as philosophy or cognitive sciences do.

If we do not understand components, functions and relations of the real-world ecosystem, i.e., its knowledge/concept space, we cannot properly model and formalize the integrated and interoperable ecosystem we are looking for, and thus, we cannot formulate the requirements and the design for correct solutions. Furthermore, we must keep in mind that we cannot decide on the correct integration and interoperability at data level without knowing the use case-specific context, objectives or constraints. Instead, we shall do this at the real-world business system level. The reasons for the aforementioned problems and appropriate solutions are discussed in more detail in [12,13].

### 2.1. Knowledge Representation and Management

Alter defines knowledge as “a combination of instincts, ideas, rules, and procedures that guide actions and decisions” [14]. The Merriam-Webster Online Dictionary defines knowledge as “the sum of what is known: the body of truth, information, and principles acquired by mankind” [15].

According to Davenport et al., knowledge is “information combined with experience, context, interpretation, and reflection. It is a high-value form of information that is ready to apply to decisions and actions” [16].

There are different knowledge classes such as the following:

- Classification-based knowledge;
- Decision-oriented knowledge;
- Descriptive knowledge;
- Procedural knowledge;
- Reasoning knowledge;
- Assimilative knowledge.

From the modeling perspective, three levels of knowledge representation are distinguished and must be consecutively processed:

- Epistemological level (domain-specific modeling)
- Notation level (formalization, concept representation)
- Processing level (computational, implementations)

**Figure 2:**
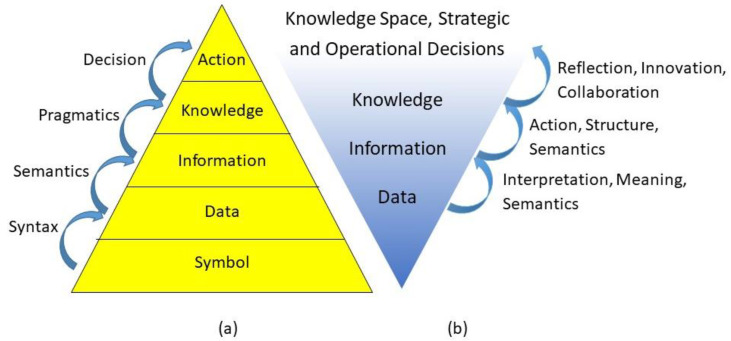
Knowledge pyramid (**a**) (after [17]) and model hierarchy (**b**) (after [18]).

Thereby, we have to distinguish different levels of systems representation and modeling: data, information, knowledge and decisions (Figure 2). A model is therefore defined as a representation of objects, properties, relations and interactions of a domain, enabling rational and active business in the represented domain.

The generalization of domain-specific epistemological models requires their transformation into a universal KR notation based on the aforementioned universal type theory and universal logics. The outcome must be validated on a real-world system and thereafter adopted if needed [19].

KR provides a theory of reasoning, composed of the following:

(a) The representation’s fundamental conception of intelligent reasoning;
(b) The set of inferences the representation sanctions (the proof theory);
(c) The set of inferences it recommends.

Furthermore, KR supports pragmatically efficient computation by properly organizing information to facilitate making the recommended inferences. Finally, KR is a medium for human expression by defining the language to represent the world.

The dynamics of knowledge creation, especially the importance of tacit knowledge and its conversion into explicit knowledge, have been analyzed by Nonaka and Takeuchi [20]. The process of converting tacit knowledge into explicit concepts through the use of abstractions, metaphors, analogies or models is called externalization. More details on KR and KM can be found in [7].

### 2.2. Language Theory and Classes of Languages

A formal language is a set of words, i.e., finite strings of letters, symbols or tokens, called the alphabet, over which the language is defined. A formal language is often defined by means of a formal grammar (also called its formation rules); accordingly, words that belong to a formal language are sometimes called well-formed words (or well-formed formulas). Formal languages do not have semantics, so they are often used as the basis for richer constructs endowed with semantics. Formal languages are also used in logic and in foundations of mathematics to represent the syntax of formal theories. Logical systems can be seen as a formal language with additional constructs, like proof calculi, which define a consequence relation.

In summary, a formal language can be given as strings

- generated by some formal grammar;
- described or matched by a particular regular expression;
- accepted by some automaton, such as a Turing machine or finite state automaton, for which some decision procedure (an algorithm that asks a sequence of related YES/NO questions) produces the answer YES.

**Figure 3:**
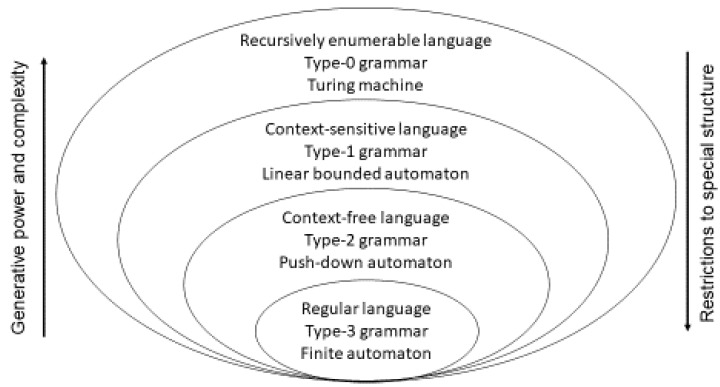
Chomsky Hierarchy Set Inclusion (after [21], modified).

Symbols, operators and interpretation theory give sequences of symbols’ *meaning* within a KR. Figure 3 classifies languages, related grammars to generate, and automata to accept them, according to the Chomsky Hierarchy Set Inclusion.

### 2.3. Representation of Knowledge-Based Ecosystems

A key parameter in choosing or creating a KR is its expressivity. The more expressive a KR, the easier and more compact it is to express a fact or element of knowledge within the semantics and grammar of that KR. However, more expressive languages are likely to require more complex logic and algorithms to construct equivalent inferences, resulting in a trade-off between expressivity and practicality [7]. A highly expressive KR is also less likely to be complete and decidable. Less-expressive KRs may be both complete and decidable [22,23]. As mentioned before, we cannot decide on the correct integration and interoperability at the data level due to its higher expressive language. Instead, we shall do this at the real-world business system level, with its less expressive and more complete and decidable languages, thereafter transforming the representations in more expressive and better-processible representation styles.

Any business system can be represented using information and communication technology (ICT) ontologies. However, the justification of the correctness and completeness of structure and behavior of the represented ecosystem can only be provided at the ecosystem’s business view using the involved domains’ ontologies. Justification of structure and behavior representation includes the representational components, their underlying concepts, their relations, but also the related constraints.

Therefore, natural languages are not only efficient in representing meaning, shared knowledge, skills, and experiences assumed. They also provide an optimum between restriction to special structure and generative power enabling the rich and nevertheless decidable representation of real-world concepts, supported of course by common sense knowledge.

**Figure 4:**
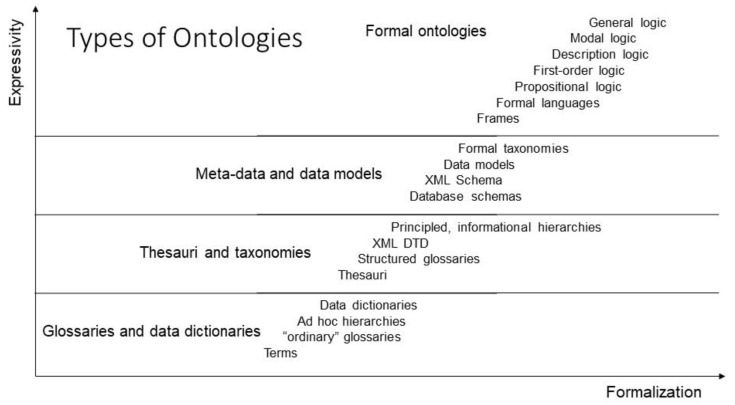
Ontology types, after [24], modified.

Knowledge can be represented at different levels of abstraction and expressivity, ranging from implicit knowledge (tacit knowledge) up to fully explicit knowledge representation, i.e., from natural language up to universal logic, using different ontology types (Figure 4).

**Figure 5:**
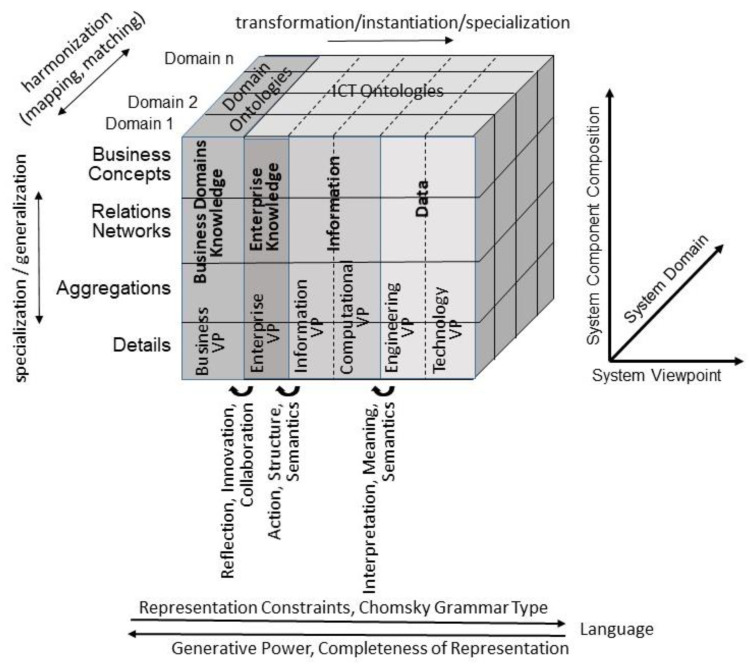
ISO 23903 mandatory framework for representing ecosystems [5].

For representing an ICT-supported ecosystem, Figure 1 must be refined including the system development process according to ISO/IEC 19746 [25]. The result is a model with the following three dimensions: system domains, system components composition (granularity) and systems viewpoints. The representation language type from natural as well as domain languages and ontologies through Business Process Modeling Language (BPML), terminologies and Unified Modeling Language (UML) up to data models and database schemas completes the latter. Meanwhile, under the lead of the first author, this model and framework has been standardized in ISO 23903:2021 [26]. Figure 5 presents the 5PM Healthcare Ecosystem model.

In order to realize ubiquitous health, intelligent and autonomous systems (AIS) are inevitable. This includes artificial intelligence (AI) and robotics [27], using machine learning, deep learning, neural networks, big data and analytics at different levels. As described in detail in [1], intelligence is a concept in cognition theory with four foundational principles: data, information, knowledge and wisdom. During investigations and observations, organs or sensors collect data as measures or symbols describing the world, establishing the structural level of intelligence. To be able to take decisions, we must transform data into information by attaching meaning to the data, establishing the semantic level of intelligence. Knowledge enables proper actions on the represented system, supervised and evaluated by wisdom, establishing the practical level of intelligence. More background information on knowledge representation and intelligence can be found in [6].

As demonstrated, highly complex, multidisciplinary, dynamic, transformed (i.e., knowledge-driven) health and social care systems must be represented and developed using a system-theoretical, architecture-centric, ontology-based and policy-driven approach. The system-theoretical considerations shall follow the white box approach [28]. Policies and related governance schemes control the behavior of the designed, finally implemented and managed 5PM ecosystem. This includes procedural requirements expressed in procedural policies, legal requirements formulated in laws and legislations including security and privacy challenges, but also the implementation of ethical and moral principles.

The ecosystem shall behave ethically, e.g., by following the Seven Principles of Public Life developed by the Committee on Standards in Public Life in Great Britain: Selflessness, Integrity, Objectivity, Accountability, Openness, Honesty and Leadership. Another Code of Conduct has been established by MCI WorldCom, Ashburn, Virginia, USA. Its guiding principles are as follows: Build Trust and Credibility, Respect for the Individual, Create a Culture of Open and Honest Communication, Set Tone on the Top, Uphold the Law, Avoid Conflicts of Interest, Set Metrics and Report Results Accurately, Promote Substance over Form, Be Loyal, and Do the Right Thing. Both ethical codes have been considered in [29]. UNESCO has established a framework for ethical AI. As a global organization covering both developed and low- and middle-income countries (LMICs), each with their economic, social and environmental challenges, the code is quite generic, covering underlying values to follow, principles to be met, as well as necessary policies. The defined values are as follows: respect, protection and promotion of human rights and fundamental freedoms and human dignity; environment and ecosystem flourishing; ensuring diversity and inclusiveness; living in peaceful, just and interconnected societies. The principles are comparable with those in other codes of conduct. An aspect frequently not explicitly declared are the necessary policies, such as ethical impact assessment, ethical governance and stewardship, data policy, development and international cooperation, environment and ecosystems, gender, culture, education and research, communication and information, economy and labor, and health and social well-being [30]. The framework was adopted on 21 November 2021 at the General Conference of the United Nations Educational, Scientific and Cultural Organization (UNESCO) in Paris.

The underlying ICT technologies shall meet the Principles for Responsible Algorithmic Systems [31] defined by the Association for Computing Machinery’s global Technology Policy Council (TPC). The stated instrumental principles are as follows:

- Legitimacy and Competency;
- Minimization of Harm;
- Security and Privacy;
- Transparency;
- Interpretability and Explainability;
- Maintainability;
- Contestability and Auditability;
- Accountability and Responsibility;
- Limitation of Environmental Impacts.

A similar set of ethical AI principles has been published by Adeola Adegunwa [32]. Besides the aforementioned principles, Adegunwa added the following ones:

- Respect for Human Autonomy;
- Fairness;
- Reliability and Safety;
- Inclusivity.

In order to put those ethical AI principles into practice, appropriate policies and procedures must be established, such as AI ethics policies, data governance procedures, algorithm accountability procedures, AI safety protocols and training and awareness programs. Thereby, a proactive approach to ethical challenges is inevitable. This implies ethical considerations in design and development, impact assessment, continuous monitoring and evaluation, inclusion of diverse perspectives as well as planning for future scenarios.

The pHealth 2021 keynote paper [1] discusses further ethical frameworks such as The Asimolar AI Principles of the Future of Life Institute [33]; the Congress Resolution Supporting the Development of Guidelines for the Ethical Development of Artificial Intelligence [34]; the BS 8611:2016 Robots and Robotic Devices Guide to the Ethical Design and Application of Robots and Robotic Systems [35]; and the OECD Principles for AI Research and Development presented at the Conference Toward AI Network Society, April 2015, in Japan [36]. A specific standard addressing the ethical challenges in designing ICT systems has been established by IEEE as IEEE 7000 [37]. This standard sets the framework for a series of IEEE standards [38], dealing with related issues such as security (IEEE 2933 [39]), privacy (IEEE 7012 [40]), and the representation of ethically driven robotics and automation systems (IEEE 7007 (IEEE 7007-2021 standard is freely available and accessible at https://ieeexplore.ieee.org/browse/standards/get-program/page/series?id=93 (18 December 2022)) [41]).

More information can be found in [13,42]. Table 5 summarizes the essence of those different ethical frameworks.

Google established the following six objectives for AI applications: be socially beneficial, avoid creating or reinforcing unfair bias, be built and tested for safety, be accountable to people, incorporate privacy design principles, and uphold high standards of scientific excellence [43].

A related and recently pushed approach addresses the so-called responsible AI. Examples can be found in [44,45,46]. Another approach is emotional AI, combining affective computing and artificial intelligence [47,48,49].

Further discussions of ethical, trustworthy, secure and safe ecosystems can be found in other papers from the authors of this paper, such as [4,42,50,51,52,53,54].

## 3. Representation of Intelligent and Ethical 5PM Ecosystems

**Figure 6:**
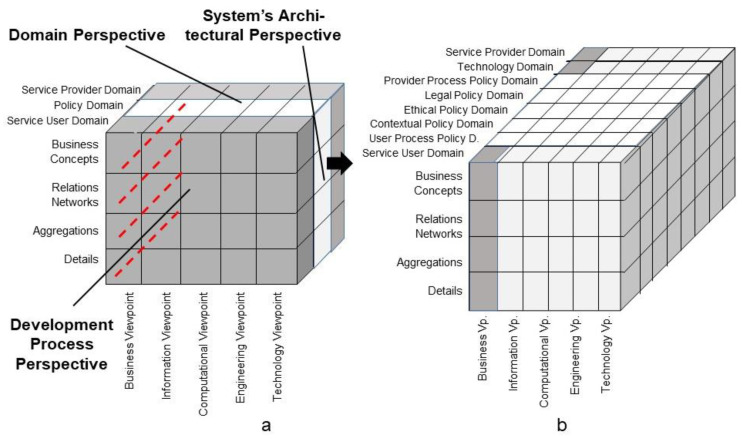
Refinement of the ISO 23903 Policy Domain.

As a starting point for designing and managing intelligent and ethical 5PM ecosystems, the domains including the related actors involved in the business system use case must be defined. The 5PM ecosystem policy domain (Figure 6a) can be refined to consider specific aspects such as the ethical policy, the legal policy, contextual policies, but also the service user’s individual policy and the service provider’s process-specific policy, as shown in Figure 6b.

**Figure 7:**
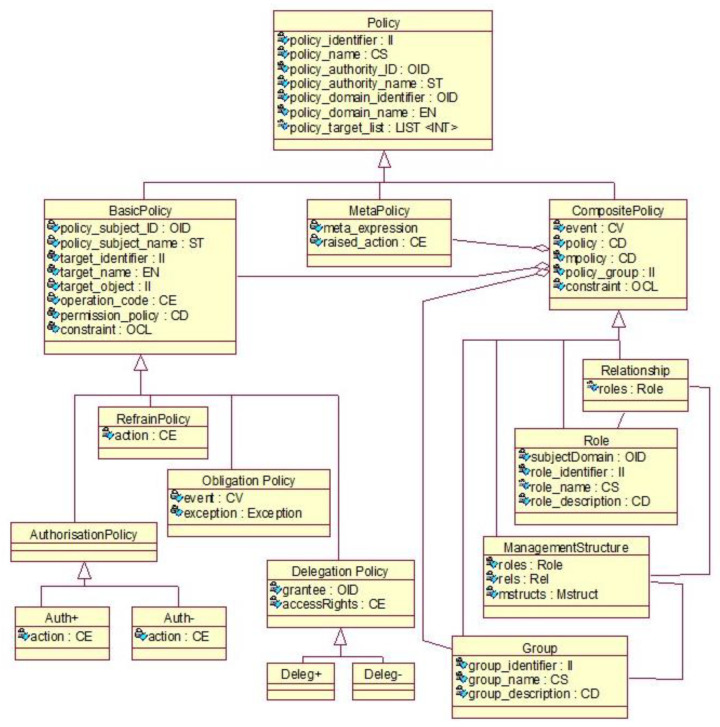
Policy ontology acc. to ISO 22600 [52].

The concepts behind the domain-specific architectural components of the business system must be represented using domain-specific languages, ontologies and methodologies. Many years ago, the first author defined a policy ontology, standardized as ISO 22600:2014 Health informatics—Privilege management and access control [55]. Figure 7 presents the ISO 22600 policy ontology.

In the next step, the sub-policy domains must be formally represented. This requires ontologies to represent the functionality or behavior of the ecosystem from the business process [56,57], the legal [58], the ethical [39] as well as the security and privacy [59,60] perspectives. Having the scope of the paper at hand in mind, in the following passages, we focus on ethical concepts including security and privacy issues.

An ontology example for legal reasoning and enforcement of security rules is PrOnto, presented in [61]. This ontology has the base components data and documents, actors and roles, processing and workflows, legal rules and deontic formula, as well as purposes and legal basis. Data categories are personal data (including pseudonymized data) and non-personal data (including anonymized data or legal person data). For more details, refer to [61]. To formally represent security requirements of ecosystems, Souag et al. defined three main dimensions and related details [60]:

- An organization with agents, assets and locations;
- Risk with severity, threat incl. threat agent, attack method and tool, vulnerability and impact;
- Treatment with security goals, requirements, criterion and control.

**Figure 8:**
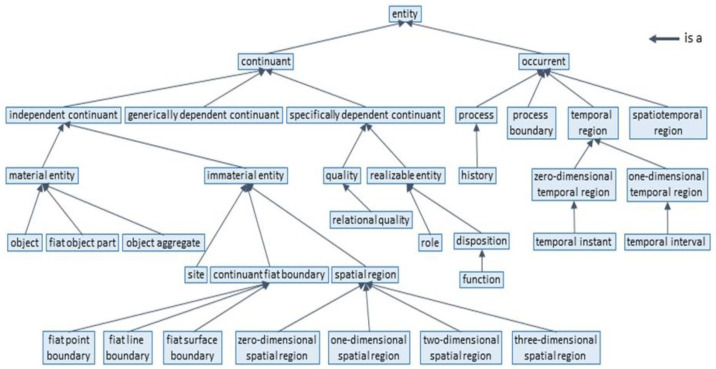
BFO is a hierarchy (after ISO/IEC 21838-2 [66]).

**Figure 9:**
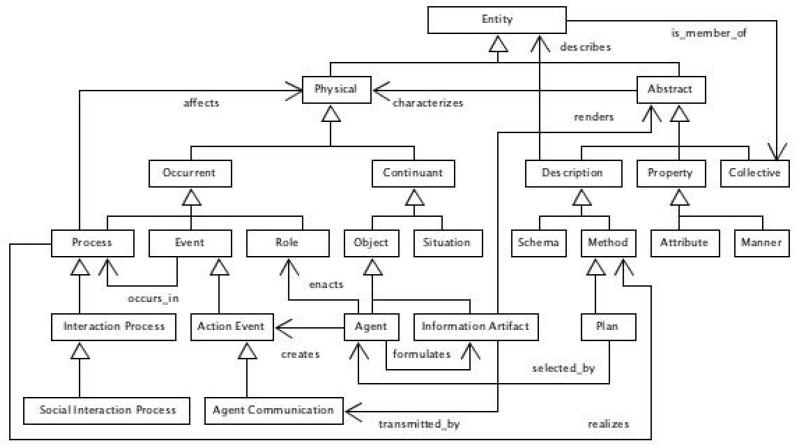
ERAS partial top-level concepts UML diagram.

The integration of ethical and trust aspects of autonomous and intelligent 5P medicine ecosystems has been developed at IEEE with a first global ontological standard for ethically driven robotics and automation systems (ERAS) [41] and is discussed in some detail in [62]. One foundational top-level model and four middle core subdomain models comprise the ERAS ontology. Each model defines respective semantic commitments using Common Logic Interface Format (CLIF) axioms [63]. The top-level ontology (TLO) was composed with concepts similar to other top-level ontologies such as SUMO [64], UFO [65] and BFO [66] to facilitate feasible alignment and harmonization. The ISO/IEC 21383:2020 Basic Formal Ontology (BFO) [66] conceptual taxonomy is shown in Figure 8, and the IEEE 7007:2021 ERAS TLO concepts and relationships are shown in Figure 9.

**Figure 10:**
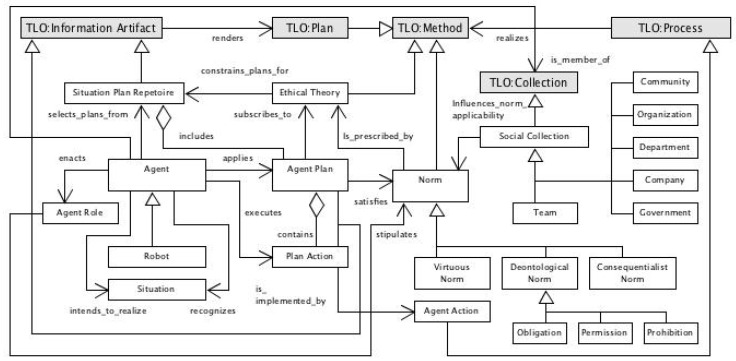
ERAS partial norms and ethical principles.

**Figure 11:**
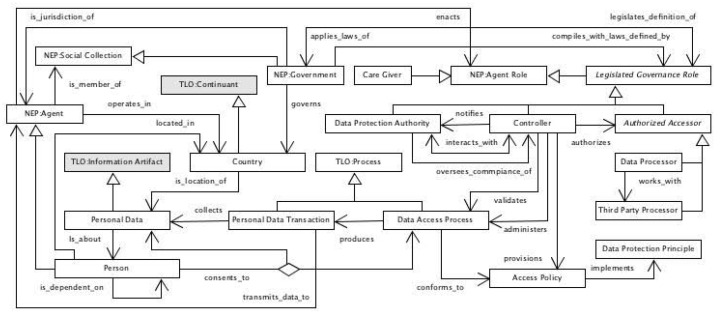
ERAS partial data privacy and protection UML diagram.

IEEE 7007:2021 specifies models and logical representations for the sub-domains Norms and Ethical Principles (NEP), Data Protection and Privacy (DPP), Transparency and Accountability (TA) and Ethical Violation Management (EVM). NEP conceptualizes principles involved in agent ethical behavior such as norms, plans and actions. DPP formalizes concepts relating to privacy and protection of agent data. TA details behaviors involved with an explanation of the agent plans and actions. EVM formalizes the concepts involved with situations where agents fail to conform with prescribed norms associated with agent plans. Figure 10 presents the IEEE 7007 ERAS norms and ethical principles ontology as a UML diagram, while Figure 11 addresses data privacy and protection.

**Figure 12:**
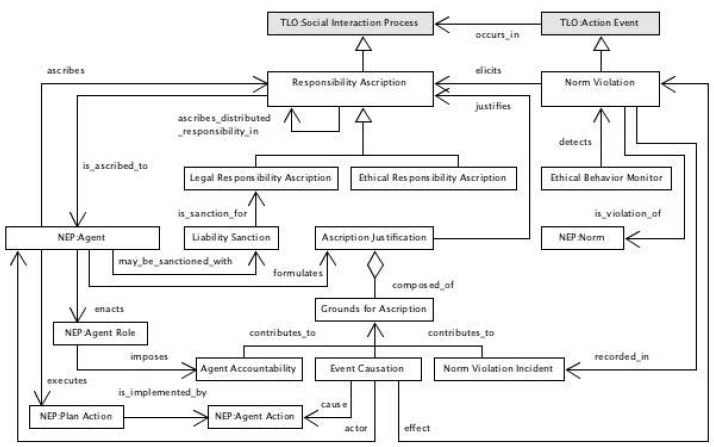
Ethical violation management UML diagram.

As mentioned before, the establishment, management and enforcement of an appropriate governance is inevitable to guarantee appropriate intentions and practices for developing and deploying advanced ecosystems regarding security, safety and privacy as well as ethical aspects. Those governance schemes must be properly and formally represented. While the security and privacy aspects have been addressed by the policy ontology, the ontology for managing ethical violations to realize responsible AI and its ontology is shown in Figure 12.

More information and complete UML diagrams for the ERAS ontology are available for free from the IEEE GET program.

There are also approaches to modelling such a system not from an information model perspective using UML, but representing the system with mathematical and statistical expressions. An example that deals with modeling morality using prospective logic can be found in [67].

## 4. Conclusions

This paper addressed the challenges in designing and managing knowledge-based, policy-driven, but also ethical 5PM ecosystems. In that context, we had to formally represent the knowledge spaces of all contributing domains using approved ontologies, languages and methodologies. For clinical domains, there are several specialized sub-domain (disciplinary) ontologies. When such ontologies are missing, we can derive related ontologies from the ISO/IEC 21838 Top-level ontologies standard [66]. Referring to international standards, we exemplified the concepts for managing the behavior of health ecosystem through related policies in an ethical way.

This paper presents a foundational, sophisticated and therefore future-proof approach to advanced ecosystems. Meanwhile, the provided theoretical considerations have been widely deployed in practical projects and international standards for designing and implementing interoperable and integrable transformed health ecosystems with the essential involvement of the authors. The first author was, e.g., strongly involved in the specification of the personal privacy consent defined in IEEE 7012 [40] or the HL7 Privacy and Security Logical Data Model, Release 1, June 2021 [68]. Both standards are based on ISO 23903:2021 [26], that way guaranteeing a correct and consistent model of the ecosystem, its domains and the development process. More details about those solutions and related standards will be presented in another paper published in the MDPI JPM pHealth 2022 Special Issue [69].

Innovations in science and technology can improve the delivery of health and social services, but they can also pose risks to global health, e.g., by strengthening the digital divide between rich and low- and middle-income countries. Therefore, they are always bound to new social, moral and ethical challenges [70]. Hereby, objectives, basic principles, limitations, etc., must be carefully considered and defined in their economic, social, political and environmental contexts. A deeper discussion is provided in [1].

## Figures and Tables

**Table 1:** Organizational paradigm changes in the evolution of 5P medicine (after [4], modified).

Care Type	Organization, Service Provision	Actors	Services	Target
Phenomenological medicine	Organization centered, Local services	Regulated professionals	Domain-specific general services	Humanity
Evidence-based medicine	Organization centered, Local services	Regulated professionals	Domain specific, group specific services	Disease-specifically defined group
Person-centered medicine	Cross-organizational local services	Regulated professionals	Multiple domains’ services	Individual
Personalized medicine	Distributed local and remote services	Regulated and non-regulated, professionals, laymen, technical systems	Multiple domains’ services, Telemedicine	Individual in personal disposition
Systems medicine	Distributed cross-domain services, Smart Healthcare	Regulated and non-regulated, professionals, laymen, technical systems	Cross-domain services, Consumerism, Telemedicine	Individual in personal, environmental, social, occupational and behavioral context
Ubiquitous personal health	Ubiquitous services	Regulated and non-regulated, professionals, laymen, technical systems	Integrated services, Consumerism, Ubiquitous medicine	Individual under comprehensive focus

**Table 2:** Methodological paradigm changes in the evolution of 5P medicine (after [5], modified).

Care Type	Way of Practicing	Justification	Representation Style	Electronic Comm./Coop	Standards
Phenomenological medicine	Observation	Pattern recognition	Data	Local data repository (inside the unit)	Data standards
Evidence-based medicine	Observation with objective evaluation	Statistical justification, group-specific treatment outcome	Information	Central data repositories	Information standards
Person-centered medicine	Managed care	Process mgmt., best medical practice guidelines	Agreed terminology, DMP best practice guidelines	Cross-organizational business process	Terminology standards; Process standards
Personalized medicine	Considering the pathology of disease	Clinically justified individual status and context	Disciplinary concepts in a situational context	Knowledge management	Domain ontology standards
Systems medicine	Understanding the pathology of disease	Scientifically justified individual status and context	Multidisciplinary concepts in a comprehensive context	Knowledge space management	Multiple ontologies guided by standards in top-level ontologies
Ubiquitous personal health		Dynamically and scientifically justified individual status			

**Table 3:** Transformed health ecosystems’ objectives and characteristics as well as methodologies for meeting them, after [6].

Objective	Characteristics	Methodologies/Technologies
Provision of health services everywhere anytime	Openness Distribution Mobility Pervasiveness Ubiquity	Wearable and implantable sensors and actuators Pervasive sensor, actuator and network connectivity Embedded intelligence Context awareness
Individualization of the system according to status, context, needs, expectations, wishes, environments, etc., of the subject of care	Flexibility Scalability Cognition Affect and Behavior Autonomy Adaptability Self-organization Subject of care involvement Subject of care centration	Personal and environmental data integration and analytics Service integration Context awareness Knowledge integration Process and decision intelligence Presentation layer for all actors
Integration of different actors from different disciplines/do-mains (incl. the participation/empowerment of the subject of care), using their own languages, methodologies, terminologies, ontologies, thereby meeting any behavioral aspects, rules and regulations	Architectural framework End-user interoperability Management and harmonization of multiple domains including policy domains	Terminology and ontology management and harmonization Knowledge harmonization Language transformation/translation
Usability and acceptability of pHealth solutions	Preparedness of the individual subject of care Security, Privacy and Trust Framework Consumerization Subject of care empowerment Subject of care as manager Information-based assessment and selection of services, service quality and safety as well as trustworthiness Lifestyle improvement and Ambient Assisted Living (AAL) services	Tool-based ontology management Individual terminologies Individual ontologies Tool-based enhancement of individual knowledge and skills Human-centered design of solutions User Experience Evaluation Trust calculation services

**Table 4:** Technologies and methodologies for transforming health ecosystems (after [7], modified).

Mobile technologies, biotechnologies, nano- and molecular technologies Big data and business analytics Integration of analytics and apps Assisting technologies → robotics, autonomous systems Natural Language Processing → text analytics → intelligent media analytics Conceptualization → knowledge management (KM) and knowledge representation (KR) → artificial intelligence (AI) → artificial common (general) intelligence → intelligent autonomous systems Security and privacy, governance, ethical challenges, education → ethical AI principles Cloud computing, cognitive computing, social business	Edge computing as a “family of technologies that distributes data and services where they best optimize outcomes in a growing set of connected assets ” [8] Virtual reality and augmented reality, thereby blurring “the boundaries between the physical and digital worlds” [9] Creation of IoT platforms and APP-based ecosystems Patient-generated health data ecosystem → multiple, dynamic policies Web content management → digital experience management Databases → NoSQL technologies → data warehouses → Graph DBs → data lakes EHR extension with genomic data Specifications → implementation → tooling → testing → certification

**Table 5:** Common A/IS principles proposed by different organizations (after [1]).

Guideline Originator	Transparency	Accountability	Controllability	Security	Value Orientation Ethics	Privacy	Safety	Risk	User Assistance
OECD	x	x	x	x	x	x	x		x
IEEE	x	x	x		x	x		x	x
Asilomar	x	x	x	x	x	x	x	x	
US Congress	x	x	x	x		x	x		x
World Economic Forum				x	x			x	

## Data Availability

The original contributions presented in the study are included in the article. Further inquiries can be directed to the corresponding author.
